# Experimental Researches

**Published:** 1855-01

**Authors:** 


### BOOK NOTICES.

*Experimental Researches*, illustrative of the Functional Oneness, Unity, and Diffusion of Nervous Action; in opposition to the Anatomical Assumption, of four sets of nerves, and a fourfold set of functions, and transmitted impressions; with a brief exposition of the Philosophy of Vivisection, and of Sensation. By BENNET DOWLER, M. D, of New Orleans.

The above is the title to a pamphlet of 66 pages, republished from the New Orleans Medical and Surgical *Journal;* and though recently received by us, it bears date in 1851. The nature and objects of the essay are sufficiently indicated by its title. It contains several of Dr. Dowler's well known experiments on the alligator, accompanied by criticisms on the generally received doctrines in relations to the functions of the nervous system.

We have read this paper carefully for the purpose of ascertaining what the real sentiments of the author are, and how far these sentiments are legitimate deductions from his experiments. We say that we have read the paper carefully, because there is in it a certain kind of vagueness and want of arrangement, which makes obscure the meaning of the author, and very much diminishes the satisfaction derived from a perusal of his work. He strikes boldly at existing opinions, and by his experiments produces results well calculated to shake our confidence in many things which have been regarded as settled physiological truths, and yet he fails to afford us a foundation for anything more satisfactory in their place. By his experiments on the alligator, he has shown conclusively enough that, after decapitation, and even after destruction of both brain and a large part of the spinal cord, the headless trunk and limbs are capable of making co-ordinate and intelligent movements.

From these experiments he is led to deny, first: That there is any such quadruple division of the nervous system, either anatomically or physiologically, as claimed by Sir Charles Bell and Marshall Hall. He denies the existence of sufficient facts to show that there are distinct nerve filaments for ordinary sensation and motion, and involuntary sensation with reflex motion; but claims the unity or oneness, in function, of the whole cerebro spinal nervous matter.

Second: He denies the doctrine of a central or common sensorium, to which all impressions are transmitted to be recognized; claiming that it is *diffused* throughout the whole nervous system; and consequently that the nervous cords are not transmitting in their function but directly sensuous in every part where impressions are made. Hence he says:

"The doctrine of an exclusive sensorium—one restricted wholly to a spot in the brain, contradicts not only the universal intuition or experience of mankind, but is self contradictory; for sensation can have no existence in time and space, only so far as it is *felt*; it exists only at the time when, and the place where, it is cognized. At all other times and places it has no existence whatever. The pain of a whitlow is felt in the finger, and not in the optic thalami or crus cerebri."

Again he says, "the doctrine of a diffused sensorium, is an intuitive conviction; based on consciousness, at least it is such, with all not educated in a different belief."

And again: "The rejection of a *diffused sensorium*, and the general admission of one exclusively central, are, probably, in a considerable degree owing to the psychological, or rather the theoretical difficulties with which education has environed these views, in assuming them to be incompatible with the supposed oneness of life, personal identity, the union of volition and sensation."

In contending that the nerves are not mere conducting cords, but possessed of perceptive or cognate qualities, Dr. Dowler uses the following language, viz.:

"Physiologists who reduce the nerves to the condition of mere conductors to and from a central, yet unknown, unconscious spot, called the sensorium, in some unrevealed part of the brain, ought not to regard themselves as honoring the nervous system, any more than those who are willing to allow these supposed conductors a *share of sensational cognition.*" * * * "Sensation is not produced by the intermediary agents called transmitted impressions or ideas, but is an intuitively felt relation, between the *Me* and the not *Me*." * * * * * "When an object is touched, the result is a felt relation directly between the subject and object, *not* between the subject and a *mere representation* of the object."

These extracts are sufficient to show the correctness of the two propositions already given as embodying the peculiar sentiments of the author. To sustain these views, he adduces his experiments on the Alligator, already alluded to; the fact that some of the lowest species of animals may be cut into many pieces, and each piece not only retain its vitality but increase in bulk, and exercise all the functions of the whole animal; the fact that under certain circumstances muscular contractions may be induced several hours after death; and finally that persons with extensive injuries of the brain and acephalous fetuses have given proof of both perception and intelligent movements. It may be well briefly to examine these several classes of facts and see how far they warrant the conclusions of the author. That several of the lower species of animals possess only the most simple or rudimentary form of a nervous system, without any central organ or cephalic ganglia, is a well known fact. And it is doubtless true that in such, each portion of the nervous matter is independent of the rest, and equally independent of any common sensorium. But it is equally true, that some of these animals have no central or special organs of respiration, circulation, or digestion—no lungs, no heart, and no stomach; but each part of their organization imbibes its own air and nutriment and performs its own excretion. Hence each part is capable of an independent life, and when separated from the rest will often retain its vitality and continue its growth. But to allege the apparent independent vitality of each part of these primitive types of the animal kingdom, as evidence of a "diffused sensorium" in the higher classes or in man, would be just as absurd as to allege the same fact in proof that man had a "diffused" digestion or respiration.

The facts in reference to the organizations are not analagous, and consequently do not admit of an analogical deduction.

To invalidate the inferences drawn from experiments, by which muscular contractions have been found to follow pinching or irritation of the anterior roots of the spinal nerves; and by which the filaments composing these have been considered as the peculiar nerves of *motion*, Dr. Dowler adduces the results of many experiments to show that muscular contraction is equally produced, and even in a much greater degree, by blows on the muscles after death, or after separation from the body as in amputated limbs, and sometimes spontaneously after death from certain diseases like cholera. Granting both the correctness and the physiological importance of these experiments and observations, still they have no bearing on the question raised by the author. Had he wished to illustrate the truth of the old Hallerian doctrine, that the muscles contain an inherent property of irritability or contractility, independent of the nervous system, his experiments by which contractions were induced by blows, electricity, &c., after the functions of the brain and nervous system had ceased, would have been directly and highly appropriate. But in the application of the facts made by him, he has confounded *contractibility itself* with the *excitors* of contractions; two things essentially distinct. The muscular structure doubtless contains an inherent property of contractibility; and while it retains this it is capable of contracting on the application of a certain stimulus or excitor, no matter what may be the condition of the nervous system. Hence if blows or galvanic currents, &c., induce contractions, it simply proves that these agencies may, under certain circumstances, become *excitors* of muscular contraction; but it by no means proves that such agencies are the *usual* excitors, and much less that other agents may not act as much more *efficient excitors*, by exerting an influence through the nervous cords distributed on the muscles. On the contrary Dr. Dowler's own experiments on the alligator demonstrate, many times, the power of exciting muscular contractions by impressions transmitted through the nervous cords.—All that can be legitimately claimed, then, is that impressions through the nervous cords are not the *only* excitors of muscular contractions; but that they are the *usual normal* excitors, is in no wise d sproved by anything in the essay before us. Again, in his anxiety to establish the doctrine, that the nervous cords are not merely *transmitting* in their function, but themselves the veritable seat of perception or "sensational cognition," Dr, Dowler quotes as the opinion of Sir Charles Bell, the statement that "loss of power in consequence of tying a nerve, was not owing to compression of the tubes of the nerve, but to the obstruction of blood vessels." And after alluding to the complete insensibility produced by the stagnation of blood in the capillaries, he gravely asserts that, "these facts show, that the non-circulation of the blood, produces a loss of motion and sensation, rivaling in instantaneousness and completeness the division of the nerves, which latter operation constitutes the *experimentum crucis* of neurological dynamists." Here again, the author is right in his facts, but wrong in the inference he would have the reader draw from them. He seems not to remember that three distinct things are essential to the action of a nerve of ordinary sensation, viz: susceptibility in the nerve itself, continuity of its fibres, and an object to make an impression. The first may be suspended or destroyed by the direct application of chloroform, or hydrocianic and carbonic acids, or by cutting off entirely the supply of arterial blood; and while the susceptibility thus remains suspended no action can be excited in the nerve. This, however, furnishes not the slightest proof that the nerve, in its normal state, is not transmitting or conducting in its function. But if we leave the supply of blood to the nerve unobstructed and the susceptibility of its distal or peripheral extremity unimpaired, and find by interrupting the continuity of its fibres in a distant part of their course, that its action is entirely suspended and all sensation through it cut off, it does furnish proof, positive and clear, that the nervous cord is a medium of communication between the surface and some central point within. So far from proving that the nervous cords are not *conductors* of impressions, or that sensational cognition takes place in the extremity of the nerve where the impression is made, all the facts adduced by Dr Dowler simply show that the action of a nerve may be suspended by suspending its elementary susceptibility; a fact long well known to every enlightened physiologist. But our comments are extending too far and we must hasten them to a termination.—

Dr. Dowler's experiments on the alligator have clearly demonstrated that it is one of those cold-blooded animals which possess great tenacity of life ; that the several parts of its nervous system are apparently more independent of each other, and retain their susceptibility longer after mutilation than any of the higher orders of warm-blooded animals ; but we think they fall far short of establishing the proposition that, in man or any of the higher orders of animals the "*sensorium is diffused*" throughout the nervous system, or that "sensational cognition" actually takes place at the peripheral extremity of the nerve without transmission to the brain or the nervous centres. The brief criticisms in which we have indulged, have not been intended to disparage the interest or value of Dr. Dowler's experimental and physiological researches. The latter have given him a deservedly high reputation in the scientific world, and we hope he may live to make many more additions to the sum of human knowledge as well as to the reputation of the profession to which he belongs. We believe there is much yet to be learned in relation to the functions of the nervous system. That the doctrines of Marshall Hall and his followers are unnecessarily complex and unsupported by facts, we are well assured. We recorded our decided dissent from his views, in an article published in the transactions of the Medical Society of the State of New York, so long ago as 1842 ; and all our subsequent observations have only confirmed the views then expressed. D.

